# Synergistic vesicle-vector systems for targeted delivery

**DOI:** 10.1186/s12951-023-02275-6

**Published:** 2024-01-03

**Authors:** Christine Ardelle Marquez, Cho-Im Oh, Gna Ahn, Woo-Ri Shin, Yang-Hoon Kim, Ji-Young Ahn

**Affiliations:** 1 Department of Microbiology, Chungbuk National University, 1 Chungdae-Ro, Seowon-Gu, Cheongju, 28644 Republic of Korea; 2 Center for Ecology and Environmental Toxicology, Chungbuk National University, Cheongju, 28644 Republic of Korea; 3 Department of Bioengineering, University of Pennsylvania, 210 S 33rd St, Philadelphia, PA 19104 USA

**Keywords:** Vesicle-vector system (VVS), Targeted delivery, Drug delivery system (DDS), Extracellular vesicle (EV), Liposome, Exosome

## Abstract

With the immense progress in drug delivery systems (DDS) and the rise of nanotechnology, challenges such as target specificity remain. The vesicle-vector system (VVS) is a delivery system that uses lipid-based vesicles as vectors for a targeted drug delivery. When modified with target-probing materials, these vesicles become powerful vectors for drug delivery with high target specificity. In this review, we discuss three general types of VVS based on different modification strategies: (1) vesicle-probes; (2) vesicle-vesicles; and (3) genetically engineered vesicles. The synthesis of each VVS type and their corresponding properties that are advantageous for targeted drug delivery, are also highlighted. The applications, challenges, and limitations of VVS are briefly examined. Finally, we share a number of insights and perspectives regarding the future of VVS as a targeted drug delivery system at the nanoscale.

**Graphical Abstract:**

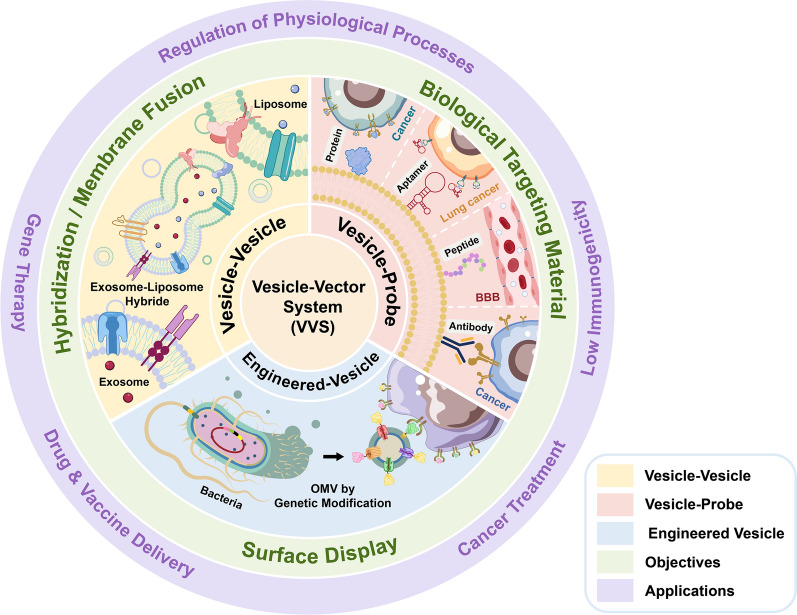

## Introduction

Drug delivery systems (DDS) refer to the approaches, formulations, manufacturing techniques, storage systems, and technologies used to transport drugs into and throughout the body. Targeted drug delivery (TDD) is the delivery of a drug to a target site with little or no effect on nontargeted cells or tissues. TDD has grown in popularity over the years due to its potential implications in the treatment of chronic diseases, including cancer, neurodegenerative diseases, and genetically acquired illnesses.

Nanotechnology has since improved drug delivery. To date, drug delivery vehicles such as polymeric micelles, liposomes, and polymeric nanoparticles have been studied as ideal drug delivery vehicles to enhance target specificity [1]. However, drugs pass through complex pathways and must overcome multiple barriers during transport to reach their target. In most cases, drugs end up interacting with multiple targets (e.g., tissues or organs), often resulting in adverse side effects. Unfortunately, delivering therapeutic materials to a targeted location remains a challenge regardless of its obvious merits.

The use of lipid-based nanovesicles as vectors for targeted drug delivery has been under development for over a decade [2]. In this review, we propose the term vesicle-vector system (VVS), a combination of the terms “vesicle” and “vector”, which pertains to the use of lipid-based nanovesicles as vectors or carriers for the delivery of therapeutic materials in a targeted manner. The VVS can be applied comprehensively in fields such as medicine, biology, chemistry, and engineering. To date, a variety of nanoparticles (NP) have been used as vectors to carry various therapeutics. These include polymeric (dendrimers, polymersomes, polymer micelles, and nanospheres), inorganic (silica NP, quantum dots, iron oxide NP, and gold NP), and lipid-based (liposomes, lipid NP, and emulsions) nanoparticles [3]. This review highlights the use of modified, lipid-based, naturally-derived or synthetically-produced nanovesicles (extracellular vesicles and liposomes, respectively) as vectors or drug carriers specifically for targeted delivery applications.

**Fig. 1:**
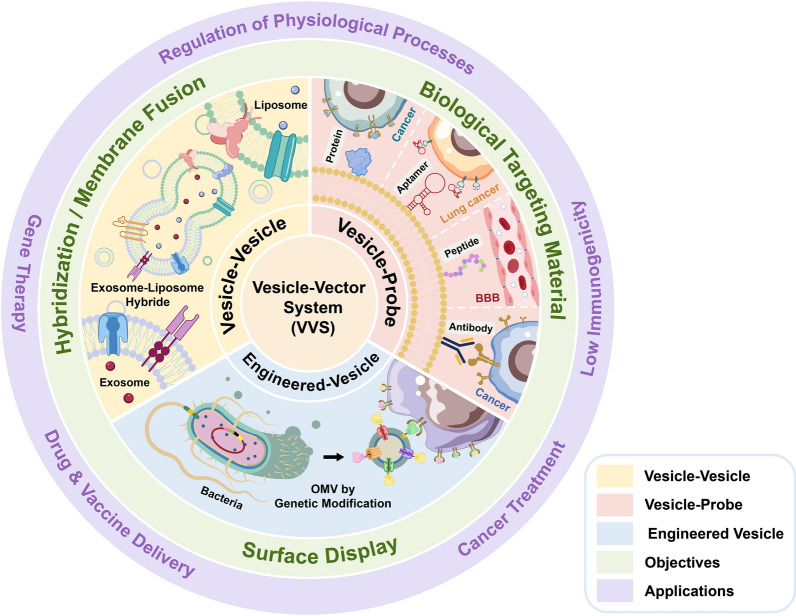
The Vesicle-Vector System. The three primary types include the vesicle-probe, vesicle-vesicle, and the genetically engineered vesicle vector systems

Here we categorized VVS into three primary types—vesicle-probe, vesicle-vesicle, and genetically engineered vesicles (Fig. 1)—based on the different conjugation techniques that will be highlighted in this review. Briefly, the vesicle-probe vector system is a lipid-based vesicle conjugated with various bioactive probing materials for targeted delivery. Polymers include a variety of proteins, peptides, antibodies, and aptamers. The vesicle-vesicle hybrid vector system is a hybrid of two types of vesicles (e.g. exosomes and liposomes, exosomes, and bacterial OMV). This type of VVS takes advantage of both natural- and synthetically-derived vesicles to achieve targeted delivery. Finally, genetically engineered VVS are based on vesicles (e.g. bacterial outer membrane vesicles) whose parent cells are genetically modified to express certain proteins (or other molecules with targeting properties) via direct or indirect cell surface display methods.

We aim to discuss various types of vesicle-vector systems (VVS), their advantages in targeted delivery, and specific conjugation techniques and methods. We will also provide some insights into how these modifications improve the target specificity of VVS as a delivery system for various cargoes such as drugs and genetic materials. Challenges, limitations, and perspectives for future studies on VVS are also reviewed.

## Vesicle types

Vesicles used in various VVS are typically divided into two types that include (1) synthetically produced vesicles such as liposomes and polymeric micelles and (2) biologically sourced vesicles such as extracellular vesicles (EV), including exosomes, bacterial outer membrane vesicles (OMV), endosomes, and vacuoles. The former has been the most studied in terms of its application in drug delivery due to its simple preparation, low cost, and overall safety [4]. However, as the functions of EVs in cell-to-cell communication have come to light, it is hypothesized that the use of EVs is potentially more advantageous than the use of liposomes in terms of targeted drug delivery due to EV’s intrinsic properties.

Each type of vesicle offers different advantages for targeted delivery. These characteristics are discussed in the following section. Table 1 presents a comparison of the lipid composition and marker molecules for each vesicle type.

**Table 1:** Lipid composition and marker molecules of liposomes and extracellular vesicles

	*Liposome*		Ref	*Extracellular Vesicle*		Refs
Lipid components	Natural phospholipids	Phosphatidylcholine, phosphatidylethanolamine, phosphatidylserine, phosphatidylinositol, phosphatidylglycerol, phosphatidic acid	[33]	*Mammalian EV (Exosome)*	Sphingomyelin, phosphatidylcholine (PC), cholesterol, phosphatidylserine, phosphatidylethanolamine	[34]
	Synthetic phospholipids	DOPS / PEG-DSPE, DOPG, DOPE, DOTAP, HSPC, DOPC, POPC, DMPC	[35]	*Bacterial OMV*	Phosphatidic acid (PA), Phosphatidylinositol (PI), Phosphatidylcholine (PC), Phosphatidylglycerol (PG), Phosphatidylethanolamine (PE)	[36]
	Stearic acid-based synthetic phospholipids	DSPE, DSPA, DSPG, DSPC	[37]	*Plant EV*	Phosphatidic Acid (PA), Phosphatidylethanolamine (PE), Phosphatidylcholine (PC)	[22]
	Palmitic acid-based synthetic phospholipids	DPPE, DPPA, DPPG, DPPC	[38]	*Yeast vacuole*	Phosphatidylcholine, phosphatidylethanolamine, phosphatidylinositol, phosphatidylserine,	[31]
Markers	None			*Mammalian EV (Exosome)*	CD9, CD81, CD63, flotillin, TSG101	[39]
				*Bacterial OMV*	OmpA porin protein (*E. coli*), Serralysin (*S. marcescens*)	[30, 36, 40]
				*Plant EV*	Tet8, PEN1, Ole e1, Ole e11, Ole e12	[41]
				*Yeast vacuole*	Yck3, Env7	[42]

### Liposomes

Liposomes are artificially produced spherical vesicles with sizes ranging from 30 nm to several micrometers and are composed of one or more phospholipid bilayers [5]. Their lipid bilayers are oriented such that the polar head groups face the interior and exterior sides, thus possessing both a hydrophobic center and hydrophilic space within the lipid bilayer. This structure makes liposomes ideal carriers of both hydrophobic and hydrophilic drugs. Liposomes are also easier to prepare than EVs. They facilitate intracellular drug delivery (of anticancer agents) and prolong the retention time of the encapsulated payload in target cells. By enhancing the pharmacokinetic profile and pharmacological qualities, liposomes can be extremely helpful in addressing the problems associated with the off-target effects of non-targeted delivery [6].

Due to their biocompatibility, low toxicity, ability to trap both hydrophilic and lipophilic drugs, and site-specific drug delivery to tumor tissues, liposomes exhibit increased rates both as an experimental system and commercially as a drug delivery system [5]. To date, liposomes remain the most researched and approved drug delivery system for clinical use [7]. Some of the most common liposome preparation methods include mechanical dispersion methods such as sonication, the freeze–thaw method, and solvent dispersion methods such as solvent vaporization and ethanol injection [5].

However, without modifications, liposomes may exhibit low target specificity, low stability, high biodegradability, and high blood clearance, thus making them difficult to use for targeted delivery [8]. Furthermore, conventional liposomes are easily degraded by pH, enzymes, and the immune system in biological environments. Under acidic pH conditions, liposomes are easily decomposed, and upon reaching the digestive system, they can be degraded by lipases [8]. These limitations can be overcome by modifying the physical and chemical attributes of liposomes via polymer conjugation, surface decoration, and hybridization with other vesicles.

### Extracellular vesicles

Extracellular vesicles (EVs) represent an umbrella term referring to the sac-like, lipid-based nanoparticles that all cells produce. EVs are secreted by most cell types through different biogenesis pathways. Depending on their parent cells, they may consist of a broad range of proteins, particles, and other molecules that ultimately define their functions and properties. They range from 30 nm to a few microns in size and are classified into several subtypes: exosomes, microvesicles, apoptotic bodies, and bacterial outer membrane vesicles [9]. Although the distinction among EV types is not well-established, researchers have determined the hallmarks of each type in terms of biogenesis, molecular composition, and function.

It was originally established that EVs are only involved in the management of cell metabolites and wastes by packaging and releasing unwanted cellular material. Recent studies have demonstrated that EVs play a vital role in cell-to-cell communication. Since the emergence of this new information, EVs have been considered excellent candidates for vesicle-based drug delivery vectors. As both are composed of phospholipid membranes, EVs and liposomes are comparable in terms of targeted delivery. In contrast to liposomes, EVs are composed of a complex mixture of different lipids and also surface and membrane proteins. Some of these components primarily function in tissue targeting, whereas others aid in minimizing nonspecific interactions. It has been hypothesized that these distinctive protein-decorated phospholipid vesicles possess precise barcodes required to locate their targets both locally and distantly [10]. Thus, EVs are ideal vectors for targeted drug delivery. The challenge lies in the isolation and purification process. The ease of isolation and purification of EVs may vary depending upon their source. However, this typically requires a combination of chemical and physical methods such as density gradient, ultracentrifugation, and immunoaffinity capture methods [11].

#### Mammalian-derived EVs

Mammalian cell-derived EVs ranges in size typically from < 200 nm [12]. They are secreted by all cell types and have been detected in plasma, urine, semen, saliva, bronchial fluid, cerebral spinal fluid (CSF), breast milk, serum, amniotic fluid, synovial fluid, tears, lymph, bile, and gastric acid [11] and are composed of a diverse cargo of proteins, lipids, nucleic acids, and other bioactive molecules, making them capable of transmitting complex biological signals to target cells by facilitating the exchange of important signaling cargoes (e.g. hormones and growth factors) carrying DNA materials between cells [13].

Mammalian cell-derived EVs have been implicated in a wide range of physiological and pathological processes, such as immune modulation, tissue regeneration, and disease progression. They can transfer their cargo to recipient cells through various mechanisms, including endocytosis, membrane fusion, and receptor-mediated uptake. Additionally, exosomes can cross biological boundaries and target tissues or cells (particularly when they originate from autologous sources) and can cross the blood–brain barrier. Manca *et.al* reported that exosomes isolated from bovine milk deliver protein and RNA cargoes to the brain [14].

The ability of these EVs to encapsulate and deliver specific molecules, such as microRNAs or growth factors, holds tremendous potential for therapeutic applications, including drug delivery and regenerative medicine [15]. Unlike liposomes, mammalian EVs originate from cells, thus making them more biocompatible and less immunogenic. They are heterogeneous in nature and carry various protein markers on their surface. Previous studies have reported that mammalian EVs can interact with target cells and modify their cellular activity by delivering various mediators. Specifically, communication with target cells is achieved by transferring the constituent biomolecules of exosomes into the cytosol of the target cell, ultimately resulting in the regulation of target cell functions via receptor interaction and endocytosis [16]. For example, immune cell-derived exosomes contain regulatory proteins of the immune system, including MHC-I, MHC-II, CD86, and galectin-9, and can bind to target cells for cell–cell communication [16]. Additionally, mammalian EV membranes contain tetraspanin, a transport and functional protein. This makes mammalian EVs a promising targeted drug delivery vector that can be used to deliver genetic material for gene therapy and to deliver chemotherapeutic drugs [17]. This has prompted scientists to further study its structure and properties to exploit its functionalities and facilitate targeted drug delivery for the treatment of diseases that are otherwise difficult to target (e.g. cancer and neurodegenerative diseases).

One of the major difficulties associated with the use of mammalian EVs is their scale up production. Particulary, the isolation and purification of mammalian EVs remains challenging due to its requirement of specialized equipment (i.e. ultracentrifuge) and significantly low yield [17]. However, recent reports have revealed that mammalian EVs can be obtained not only through endogenous synthesis but also through dietary sources [18]. Therefore, to obtain an effective and practical VVS, the isolation of mammalian EVs from relatively more accessible sources such as animal milk [19, 20] can overcome this limitation. Of note, the cellular uptake or treatment of exosomes from different species has proven valuable and exerts significantly positive effects across different species (e.g. bovine milk in rat/mouse cell lines [19] and donkey milk in rat skeletal muscle cells [21].)

#### Plant-derived EVs

Unlike mammalian-derived EVs, studies examining plant-derived EVs (PDEV) are still in their infancy. PDEVs are typically similar in size to mammalian EVs and carry diverse biomolecules such as proteins, lipids, RNAs, and metabolites in their lumen and on their membrane surfaces [22]. Depending upon the plant source, plant-derived EVs exhibit specific characteristics that can be used for various applications. In particular, a growing body of research has demonstrated the unique characteristics of plant EVs that make them ideal for transporting anticancer agents, including biological compounds and medications, to cancer cells. For example, Zhang et al. isolated EVs from edible ginger and loaded them with doxorubicin (DOX) that was used for drug delivery in the context of colon cancer therapy [23]. The isolated EVs were almost circular in shape with an average size of 90 nm (ranging from 30 to 200 nm). They discovered that ginger-derived EVs (GDEV) were absorbed well by intestinal cancer cells, and this led to the targeted delivery of DOX to colon cancer tumor cells in vivo. Most notably, DOX-loaded GDEVs successfully reduced the tumor size in a colon xenograft model. These GDEVs were synthesized from ginger lipids and exhibited excellent biocompatibility.

Due to their capacity to traverse biomembranes and their biocompatibility, low toxicity, low immunogenicity, and less allergenic nature, clinical trials have demonstrated that PDEVs are potential delivery vehicles for natural chemicals to specific cell targets [24]. Target specificity will increase when further combined with bioactive molecules, including proteins, peptides, and aptamers, thus making PDEVs more suitable for targeted delivery (further detailed in Sect. "Bioactive Probes for Targeted Delivery").

#### Bacteria-derived OMVs

Bacterial outer membrane vesicles (OMVs) are lipid-based, sac-like vesicular membranes produced by Gram-negative bacteria. They range in size from 20 to 300 nm and are released during bacterial growth [25]. They are responsible for the transport of a broad range of chemically diverse cargoes, such as membrane-embedded and associated proteins, small molecules, peptidoglycans, and nucleic acids [26]. Another advantage of OMVs is their versatility in terms of the ease of genetic modification through basic molecular biology laboratory techniques like bacterial plasmid transformation. Furthermore, OMVs can be mass-produced using optimized purification methods as described by Gerritzen et al*.* [27]. Hence, OMVs, exosomes, and other mammalian cell-derived EVs are excellent candidates for targeted drug delivery.

Bacterial OMVs contain various proteins that vary depending on the source organism. For example, OMVs derived from *E. coli* are enriched in Omp porin proteins [28]. In particular, OmpA was observed to be significantly abundant, thus making it a reliable marker for *E. coli* OMV detection and characterization [29]. The extracellular protease serralysin is prevalent in OMVs produced by *Serratia marcescens* [30].

#### Others

Yeast-derived vacuoles are similar to mammalian EVs in terms of their membrane composition. However, unlike bacterial OMVs they are nonpathogenic and can be easily modified by genetic engineering [31]. Gujrati et al*.* developed a bio-inspired drug delivery system using vacuoles isolated from genetically engineered yeast cells [32]. The nano-sized vacuoles dramatically improved the ability of the drug to penetrate tumor xenografts, and this reduced tumor growth without inducing immune reactions.

## Vesicle-probe vector system

**Fig. 2:**
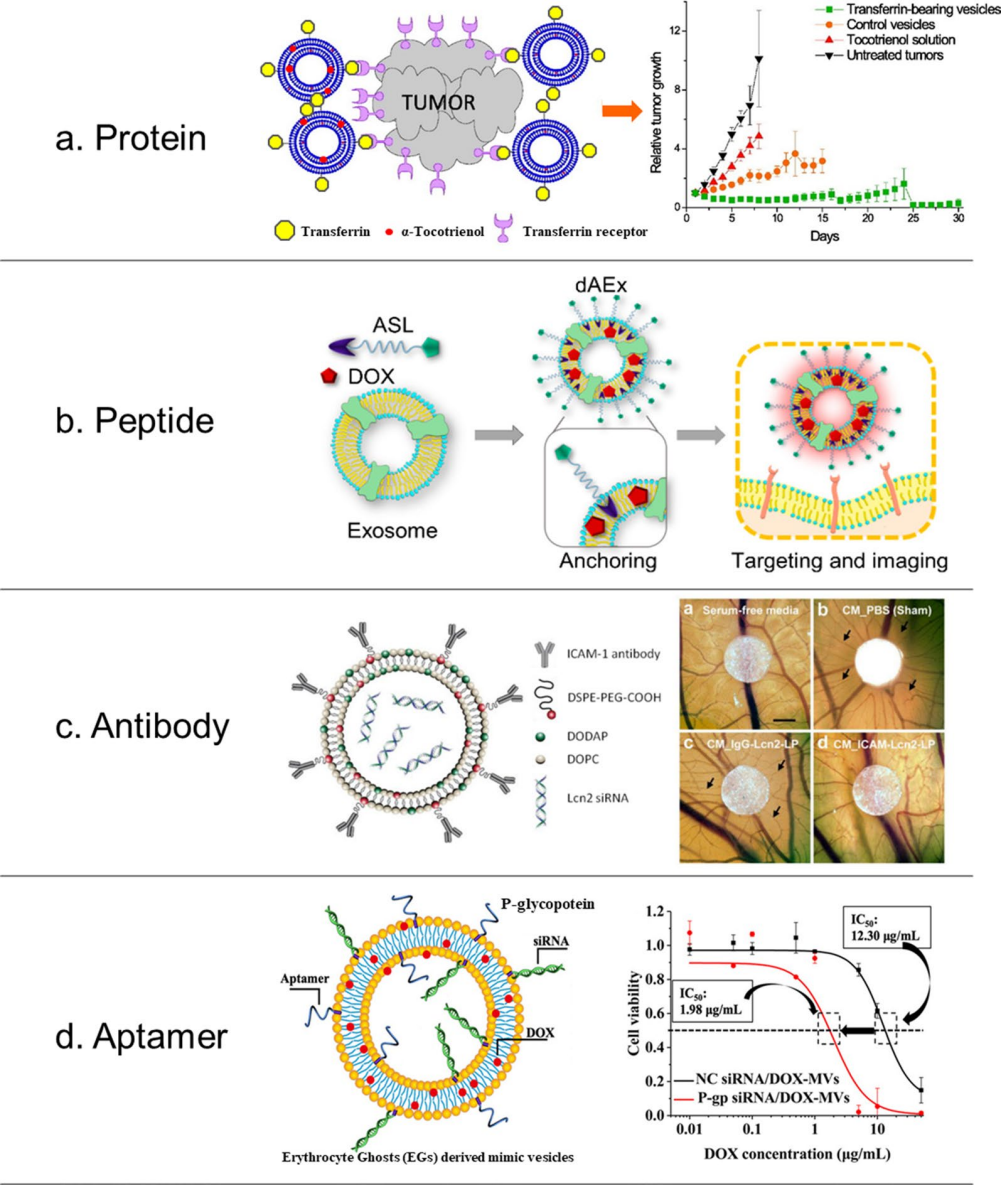
Vesicle-Probe Vector Systems **a** Multilamellar Vesicles-Protein. Transferrin-bearing multilamellar vesicles encapsulating α-T3 suppressed the growth of A431 epidermoid carcinoma and B16-F10 melanoma in vitro and in vivo. Upon intravenous administration, the modified vesicles caused tumor regression within 24 h and continued for 11 days. By day 16, the tumor began to develop again, but subsequent treatment reverted this growth right away. In comparison, other treatments failed to cause tumor regression. Reprinted from Karim et al., 2017. **b** Exosome-Peptide. Modified exosomes, composed of membrane Anchor (BODIPY)-Spacer (PEG)-targeting Ligands (cyclic RGD peptide) (ASL), enhanced the stability, target delivery, therapeutic efficacies of DOX, and added imaging capabilities to exosomes as a theranostic agent. DOX carried by the ASL exosomes were able to image the tumor specifically and effectively limit the growth of the tumor without causing any substantial side effects. Reprinted from Kang et al., 2020. **c** Liposome-Antibody. Lipocalin 2 (Lcn2) is a promising therapeutic target for breast cancer. For the specific delivery of Lcn2 siRNA, liposomes were conjugated to ICAM-1 antibody via the DSPE-PEG-COOH anchor. They were capable of targeting TNBC cells and silencing the Lcn2 gene through the inhibition in vivo of angiogenesis. Representative micrographs are presented for the reduction of blood vessel formation in the treatment of antibody conjugated liposome, ICAM-Lcn2-LP. Reprinted from Guo et al., 2016. **d** Mimic vesicles (MVs) derived from erythrocytes-Aptamer. DOX and P-glycoprotein (P-gp) siRNA was loaded onto the MVs. The MV carriers could be readily obtained through extruding erythrocyte membranes. At 5 μg/mL of DOX concentration, the viability of P-gp siRNA/DOX-MV-treated groups dropped by ≤ 10%, in comparison to the 80% reflected in NC siRNA/DOX MV-treated groups. This suggests that the aptamer conjugated MVs successfully overcame drug resistance and synergistically kill MDR tumors. Reprinted from Wang et al., 2019

For decades, phospholipid-based vesicles have become key factors in drug delivery systems. However, the innate characteristics of specific vesicle types may be inadequate for successful drug delivery. Upon preparation or isolation, vesicles are further modified to remove or add certain properties to achieve the desired results. Modification of vesicles by fusion with or addition of polymers is one of the many ways that allow researchers to add goal-specific traits to a vesicle, ultimately making it more effective in carrying out its objectives (in this case, target specificity). Functional groups situated on the lipid heads of the vesicle membrane bilayer act as anchors and allow conjugation of bioactive probes, such as proteins, peptides, antibodies, and aptamers which can improve target specificity (Fig. 2). A vesicle-probe-type VVS provides a good example.

### Bioactive probes for targeted delivery

Various methodologies exist in which different types of polymers are part of the liposome structure. Hydrophilic polymers can be added as coatings to the surfaces of vesicles via physical adsorption. Ionic polymers (such as chitosan, polylysine, and others) uses electrostatic interactions, while neutral polymers uses hydrogen bonds to attach to the surface of the vesicle [43]. These are examples of vesicle modifications via conjugation with polymers. The following section discusses the most useful bioactive probing materials that can be applied in vesicle modification for targeted drug delivery applications. Different types of bioactive probes and their corresponding interactions with vesicles, applications, and other relevant information are summarized in Table 2.

**Table 2:** Summary of proteins, peptides, and polymers used on Vesicle-Probe Hybrid VVS

Bioactive Probing Molecule		Linker Molecule	Vesicle Type	Properties	Target Site / Cell	Application/s	Refs.
Proteins and Peptides	Transferrin	GHDC	Liposomes	higher transfection efficiency; premium targeting efficacy	SMMC-7721 cells	Liver Cancer Therapy	[4]
		Dimethylsuberimidate (DMS)	Multilamellar vesicles	Targeted delivery and Vitamin E entrapment	A431 epidermoid carcinoma and B16-F10 melanoma	Cancer Therapy	[80]
	T7 (L(HAIYPRH)) peptide	DSPE-PEG	Liposomes	Targeted delivery	Transferrin-overexpressing tumor cells	Hepatocellular Carcinoma	[47]
	P1C peptide	Cell Penetrating Protein (CPP)	Liposomes	Targeted delivery	cancer cells	Cancer Therapy	[49]
	P1C peptide	DSPE-PEG	Liposomes	Targeted delivery	αvβ3-expressing tumor cells	Cancer Therapy	[50]
	Linear RGD	DSPE-PEG	Liposomes	Targeted delivery	RGD-overexpressing tumor cells	Breast Cancer Therapy	[51]
	Gastrin Releasing Peptide Receptor (GRPR) antagonist peptide	DSPE-PEG	Liposomes	Targeted delivery to GRPR expressing cells	GRPR expressing cells / Lung cancer cells	Lung Cancer Therapy	[52]
	Her2-LAMP2 fusion protein	peptide linker flexible (GGGGS)_3_ linker	Exosomes	Targeted delivery	HCT-1165FR cells	Colon cancer Therapy	[53]
	gp130 binding hydrophobic peptide (VTWTPQAWFQWV)	Dipalmitoyl-lysine-KSS-(SG)5-K3	Liposomes	Targeted delivery	human glioma U251MG cells	Brain Cancer Therapy	[54]
	Lactadherin	–	Exosomes, microvesicles	increase EV uptake by phagocytes	macrophages and microglia	Neuroinflammatory diseases	[55]
	vesicular stomatitis virus-glycoprotein(VSV-G)	A/C heterodimerizer (AP21967)	Exosomes	increase the efficiency of their invasion of target tissues	skeletal muscle cells	Muscle atrophy Therapy	[56]
Antibodies	PD-L1 monoclonal antibody	DSPE-PEG-Mal	Liposomes	Targeted delivery	Multidrug-resistant tumor cells	Gastric Cancer Therapy	[60]
	(EGa1) anti EGFR	DMPE-PEG	Exosomes	improved circulation; avoid interaction with immune cells; tumor target delivery	Tumor cells	Cancer Therapy	[61]
	anti T cell CD3 and HER2	flexible (GGGGS)_3_ linker	Exosomes	Targeted delivery	Breast cancer cells	Breast Cancer Therapy	[81]
	Anti CD3-anti EGFR complex	flexible (GGGGS)_4_ linkers	Exosomes	Targeted delivery	TNBC cells	Antitumor immunity	[82]
	Anti SIRPα-anti CD47 complex	pH-sensitive benzoic-imine bonds	Exosomes	Increased tumor phagocytosis	4T1 tumor cells	Anticancer therapy	[83]
	Anti-ICAM-1 antibody o	DSPE-PEG	Liposomes	Targeted delivery	Breast cancer cells	Breast Cancer Therapy	[62]
Aptamer	anti-EGFR aptamer (Apt)-conjugated chitosan (Apt-Cs)	Chitosan	Liposomes	superior biostability; binding specificity for EGFR-mutated cancer cells	EGFR-mutated lung cancer cells	Lung Cancer Therapy	[75]
	RNA aptamer specific to the prostate specific membrane antigen (PSMA)	DSPE-PEG2000	Liposomes	Targeted delivery, Promote tumor size regression	LNCaP cells, LNCaP xenograft mice	Prostate Cancer Therapy	[76]
	AS1411 DNA aptamer conjugated with truncated tissue factor (tTF)	Sulfo-SMCC	Liposomes	Induced intravascular thrombosis solely in tumors	tumor vascular endothelial cells	Cancer Therapy	[84]
	AS1411 aptamer	direct exosome-aptamer binding via covalent bond	Exosomes	Increased binding affinity and absorption rate	CT26 cells	Colorectal cancer Therapy	[85]
	Sgc8 aptamer (PTK7)	diacyl lipid	Exosomes	Cell-type-specific recognition, Increased cell delivery capacity of drugs	CEM cells	Cancer Therapy	[86]
	AS1411 DNA aptamer	Cholesterol linkage	Erythrocyte ghosts derived mimic vesicles	Targeted delivery	Multidrug-Resistant tumors	Cancer Therapy	[79]

#### Proteins and peptides

Proteins and peptides can be conjugated by various covalent linkages such as maleimide-thiol, peptide, sulfonyl, disulfide, and phosphatidylethanolamine-linked bonds [44]. Additionally, peptides can be adsorbed and/or interpolated onto the liposomal surface via electrostatic and/or hydrophobic interactions [45]. Furthermore, peptides can be present in several copies simultaneously to achieve a higher binding affinity, and the large specific surface area of nanostructures makes it possible to combine various peptide ligands into a single construct [46]

Most recent applications of protein/peptide modified VVS are in the field of cancer treatment (Table 2) and include transferrin [4] (Fig. 2a), T7 peptides [47], and cyclic arginylglycylaspartic acid (RGD) peptides (Arg-Gly-Asp-d-Phe-Lys) [48] (Fig. 2b). These can be used as bioactive probing materials decorated on vesicle surfaces to actively target transferrin receptor-rich liver tumor cells. Several other studies report the use of P1C peptide to decorate liposomes and target avβ3 protein and demonstrated successful targeted drug delivery of DOX to cancer cells both in vitro and in vivo [49] [50]*.* Wen et al*.* used RGD (cyclic peptide containing Arg-Gly-Asp) to target the same integrin protein avβ3 and successfully delivered shikonin (anti-tumor drug) on breast cancer cells [51]. In lung cancer cells, antagonist peptides of gastrin-releasing peptide receptors (GRPR) can be used to decorate lipid-based vesicles for targeted delivery [52].

Previous studies frequently used liposomes as lipid-based vesicles and delivery vectors. However, EVs such as exosomes are also good vesicle options for protein/peptide modification. Liang et al*.* decorated exosomes with Her2 affibodies to target Her2-expressing tumor cells in breast cancer and observed successful targeted delivery and high cellular uptake [53]. This method is also promising for the treatment of brain cancers such as glioma. The glycoprotein gp130 is overexpressed in human glioma cells, and Suga et al*.* designed a peptide, VTWTPQAWFQWV (VTW), that binds specifically to gp130 to promote the targeted delivery of drugs to glioma cells [54]. Although this study demonstrated successful targeted delivery to glioma cells in vitro, further studies are required for in vivo and clinical applications.

In addition to its applications in cancer treatment, similar successful efforts have been made in regard to the treatment of neuroinflammatory diseases [55] and muscle atrophy therapy [56]. Proteins and peptides exhibit great diversity, specificity, and targeting potential [57, 58]. These attributes have led to extensive research on proteins and peptides as new therapeutic possibilities for targeted delivery.

#### Antibodies

Antibodies are the protective proteins produced by the immune system. Vesicles can increase the efficiency of endocytosis and intracellular drug administration by conjugating with antibodies to take advantage of the interactions between the antibodies and cancer cells. The pharmacokinetic (PK) and pharmacodynamic (PD) behaviors of VVS are influenced by the surface features of the vesicle, drug-to-lipid ratios, antibody structure, affinity, and density [59].

The conjugation of antibodies to vesicles can improve the specificity of VVS. Yu et al. demonstrated the successful delivery of Paclitaxel and P-gp transport inhibitor for the synergistic treatment of multidrug-resistant gastric tumors using nanoliposomes decorated with a PD-L1 monoclonal antibody [60]. Nanobodies are a type of antibody that are small recombinant proteins that can also be used in VVS. Kooijmans et al. created nanobody-polyethylene glycol (PEG)-micelle conjugates to target tumor cells. Epidermal growth factor receptor (EGFR)-specific nanobodies were coupled with phospholipid (DMPE)-PEG derivatives. Their results revealed a significantly higher binding specificity to EGFR-overexpressing tumor cells [61]. Moreover, breast-cancer-specific immunoliposomes (intercellular adhesion molecule-1 [ICAM-1]-liposomes) promote clinical effectiveness by delivering siRNA [62] (Fig. 2c).

Multitargeted delivery can also be achieved using bispecific antibodies (BsAbs). This increases the target specificity by binding to or inhibiting two or more targets simultaneously. BsAbs possess two binding sites for distinct antigens that simultaneously recognize two different epitopes of one antigen. This increases the therapeutic potential of the cargo and minimizes the development of drug resistance that is typically observed when using single-targeted treatments [63].

#### Aptamers

Aptamers are short artificial single-stranded DNA or RNA oligonucleotides that are typically 25–90 nucleotide bases in length which can selectively bind to specific target molecules with high target affinity and specificity [64]. Over the years, aptamers have attracted attention as promising ligands due to their high affinity for a single specific target and their versatility, small size, stability, low immunogenicity, and streamlined synthesis [65]. This makes aptamers excellent candidates for bioconjugation with lipid-based vesicles [65–68].

A pertinent characteristic of aptamers that makes them suitable for target-specific delivery is the presence of guanine-rich sequences in their structure termed guanine quadruplexes (G-quadruplexes) [69]. In addition to high target affinity, these structures make aptamers highly resistant to serum nucleases and cause them to exhibit high thermal stability [70]. Aptamer-target interactions are mediated by hydrophilic bindings that include hydrogen bonding and also polar and electrostatic interactions [67]. Systematic evolution of ligands by exponential enrichment (SELEX) is an enrichment method used to produce aptamers. Using a larger oligonucleotide library, SELEX identifies the optimal aptamer candidates for targeted drug delivery [71].

Conjugation of aptamers to vesicles can be achieved using various chemical and physical methods. The post-insertion method is a simple method that allows for the efficient incorporation of targeting ligands into lipid-based vesicular membranes [72]. In this method, aptamers are conjugated after the extraction or preparation of the main vesicular body. First, the aptamers react with functional group-activated lipid chains. The resulting unstable aptamer-modified lipid micelles were then incubated with the pre-formed drug-loaded vesicles. This method is flexible and compatible with a variety of ligands, thus allowing for the fabrication of different combinational constructs.

Conversely, in synthetically prepared vesicles such as liposomes, aptamers can be incorporated via the membrane anchor method in which the aptamers are conjugated during vesicle preparation [73]. Using this method, the resulting aptamer-vesicle complex is formed in a manner such that a portion of the aptamer molecule is on the inner surface of the liposome. This could limit the internal space of the liposomes and make them susceptible to hydrolytic degradation [74]. The major drawback of this method is the exposure of aptamers to the substances and chemical solvents used in liposome preparation that may exert adverse effects on the secondary structure of the aptamers, ultimately leading to functional defects. Nonetheless, this is an established method for aptamer-vesicle conjugation.

In a study conducted by Li et al., the researchers constructed a novel aptamer-vesicle hybrid VVS for the treatment of EGFR-mutated lung cancer cells. The liposomes were conjugated with anti-EGFR aptamer (Apt)-conjugated chitosan (Cs) and were then loaded with erlotinib (Apt-CL-E) [75]. When tested in vitro on H1975 cells, erlotinib-carrying Apt-CL-E VVS demonstrated superior stability (compared to its non-decorated liposome counterparts) by preventing nanoparticle aggregation. It also exhibits binding specificity for EGFR-mutated cancer cells, and this leads to the termination of the cell cycle and apoptosis of cancer cells. Furthermore, in contrast to other RNA aptamer-mediated liposome delivery systems [76], the Apt-CL-E formulation used in this study was conjugated with DNA aptamers. DNA aptamers are easier to produce, more stable against nuclease digestion, and more easily combined with VVS [77, 78]. The cholesterol-linked DNA aptamer AS1411, combined with erythrocyte-ghost (EG) vesicles, specifically delivered siRNA and DOX to treat multidrug resistant (MDR) tumors [79] (Fig. 2d).

## Vesicle-vesicle hybrid vector system

### EV-liposome hybrids

**Fig. 3:**
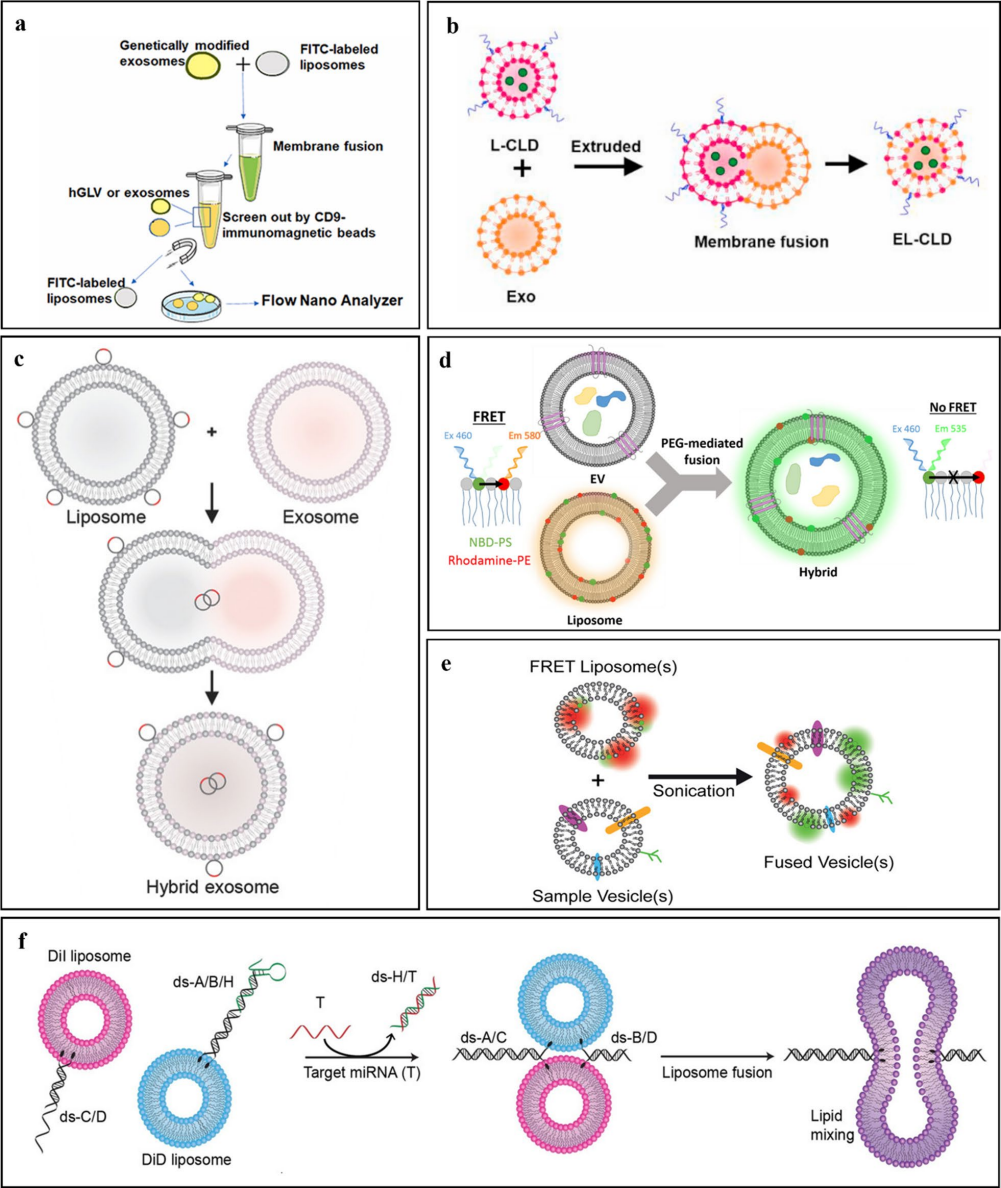
Conjugation Methods **a** Freeze–thaw Method. Reprinted from L. Cheng et al., 2021 **b** Membrane Extrusion Method. Reprinted from Sun et al., 2021 **c** Simple Incubation. Reprinted from Lin et al., 2018 **d** PEG-mediated Fusion. Reprinted from Piffoux et al., 2018 **e** Sonication. Reprinted from Thorsteinsson et al., 2020 **f** Ligand-based Incubation Method. Reprinted from Jumeaux et al., 2018

Recently, there has been interest in the development of liposome-EV hybrids for targeted drug delivery. These hybrids can be created by fusing liposomes with EVs using various conjugation techniques, such as freeze–thaw, membrane extrusion, and simple incubation (Fig. 3). Hybridization of EVs via membrane fusion with liposomes was developed as an alternative technique to various cellular uptake methods in modifying EVs. This approach was first demonstrated by Sato et al. (2016) [87] using a simple freeze–thaw method and it allowed them to optimize the intrinsic properties EVs but with decreased immunogenicity, increased colloidal stability, and improved the EV half-life in blood. In another study, Lv et al. (2020) [88] developed hybrid EV-liposome nanoparticles for drug delivery using previously reported freeze–thaw methods and showed an increased drug delivery efficiency and decreased clearance by the mononuclear phagocyte system (MPS). In addition, numerous studies have also demonstrated this principle using other conjugation techniques (Table 3).

**Table 3:** Recently developed exosome-liposome hybrid vesicle-vector system

Exosome Source/s	Liposome Preparation	Conjugation Method	Properties	Cargo	Target	Application/s	Refs.
Mouse fibroblast sarcoma-derived CMS7-wt, CMS7-HE, and Raw 264.7 macrophages	Extrusion Procedure	**Freeze–thaw method**	Enhanced cellular uptake	–	–	Drug Delivery	[87]
HUVEC, MSC, MDCK	Extrusion Method	**PEG-induced membrane fusion**	Enhanced cellular uptake; 30% more fusion efficiency than previous ref [87]	–	–	Drug Delivery	[89]
HEK293FT cells	–	**Incubation**	Delivery of CRISPRCas9 system in the mesenchymal stem cells (MSCs)	Large nucleic acids and CRISPR/Cas9 expression system	mesenchymal stem cells (MSCs)	Gene manipulation	[90]
SKOV3-CDDP cells	Thin Film Hydration and Extrusion Technique	**Sonication (ultrasound), membrane fusion and extrusion**	multitargeting capability, high drug encapsulation rate, valid drug protection and low clearance by MPS	Chemotherapy agents TP and miR497	Tumor cells	Ovarian Cancer Therapy	[91]
Fibroblast-derived (L-929 cells) via ultracentrifugation	Reverse-Phase Evaporation Method	**Membrane Fusion**	High affinity towards target cells → effective at depletion of macrophages	Clodronate (CLD)	Kupffer cells	Pulmonary Fibrosis	[92]

The composition of hybrid EVs can be designed by varying the liposome-to-EV ratio and the composition of the precursor liposomes. This is a promising bio-inspired strategy that harnesses communication and delivery systems mediated by EVs.

### Conjugation methods

#### Freeze–thaw method

The freeze–thaw method was originally utilized in the loading of therapeutic materials into lipid-based vesicles like liposomes. By briefly forming ice crystals, the membrane layer is disrupted, at which point water-soluble molecules can enter the vesicles. The same methodology can be employed in the development of liposome-exosome VVS hybrids. The frequency of freeze–thaw cycles can vary across different experimental designs. Sato et al. created liposome-exosome hybrids (with 1:1 ratio by volume) by freezing the reaction mixture in liquid nitrogen and then thawing them at room temperature for 15 min. After repeated freeze–thaw cycles, the resulting hybrid exosomes exhibited enhanced rates of cellular uptake compared to that of its non-hybridized liposome counterparts [87]. In another study, a vesicle-vesicle hybrid VVS was developed by combining genetically modified exosomes and thermosensitive liposomes to take advantage of immunotherapeutic and photothermal properties from the exosomes and liposomes, respectively. The vesicles were successfully fused using the freeze–thaw method with a fusion efficiency of 97.4% (Fig. 3a) [93].

Freeze–thaw methods are known attain considerably high fusion efficiencies, however, there are considerable drawbacks. Freeze–thaw cycles done in high frequency can compromise membrane integrity of both vesicles and it may negatively affect the stability and efficacy of the drug cargo [94].

#### Membrane extrusion method

To form hybrid vesicles using this method, both exosomes and liposomes were simultaneously extruded via membrane pores of controllable size under physical pressure. The main advantage of the membrane extrusion method over other fusion methods is the uniformity in size of the resulting vesicles. Sun et al. formed a vesicle-vesicle hybrid VVS using liposomes loaded with clodronate and exosomes obtained from fibroblasts to treat pulmonary fibrosis (Fig. 3b). The exosome-liposome mixture was vortexed, sonicated, and passed through a polycarbonate ester membrane with 400 nm and 200 nm pore size. This extrusion process was repeated ten times [92]. The pore size of the polycarbonate membrane and frequency of membrane extrusion affected the characteristics of the hybrid VVS. While membrane extrusion methods exhibit elevated fusion success rates, the mechanical stress produced throughout the extrusion procedure may compromise the membrane integrity of natural exosomes.

#### Incubation

This simple incubation method relies on the spontaneous process of membrane fusion that takes advantage of the innate physicochemical properties of both vesicle types to facilitate fusion. In incubation method, exosome-liposome VVS hybrids are formed through electrostatic or hydrophobic interactions, without the disruption of the membrane integrity or leakage of vesicle contents. Lin et al*.* created hybrid VVS by incubating HEK293FT-derived exosomes with CRISPR/Cas9-expressing liposomes at 37 °C for 12 h, (Fig. 3c). This novel study provides an interesting approach for a safe and successful delivery of CRISPR-Cas9 system in gene therapy applications [90]. Although the fusion efficiency of incubation method is notably lower than that of the freeze–thaw and membrane extrusion methods, this technique does not cause significant harm to vesicles’ membrane integrity and cargo load.

#### PEG-mediated fusion

Polyethylene glycol (PEG) has been extensively used to increase circulation time of drug-carrying liposomes. However, it has also been proven to facilitate membrane fusion between two vesicle types by intermediating close physical contact between the lipid bilayer structures and subsequently signaling the reorganization of lipid molecules. A study by Piffoux et al*.* revealed that PEG facilitates the conjugation of liposomes and exosomes (Fig. 3d). Their results indicated an enhanced fusion efficiency of at least 30% [89].

However, PEGylated VVS still has major drawbacks such as the accelerated blood clearance (ABC) phenomenon, which manifests after several doses. Various efforts were done to evade the ABC phenomenon such as the addition of polysarcosine coating [95]. Overall, PEG-mediated fusion is a notable method of membrane fusion due to PEG’s simple preparation and stability coupled with its added ability to increase circulation time of VVS in the blood.

#### Sonication

Sonication is a technique that uses sound waves to induce mechanical shear stress, impair vesicle membrane integrity, and facilitate membrane hybridization. This method was originally extensively used for liposome preparation and drug loading. However, the same principle can also be applied to vesicle hybridization. Thorsteinsson et al*.* demonstrated the successful fusion of the two liposome types using sonication (Fig. 3e) [96].

Although sonication is a powerful technique with a high fusion efficiency [96], consideration should be given to EV’ membrane integrity. The loss of intrinsic contents and biological qualities and the possibility of membrane deformation due to overheating during vesicle hybridization are key points of concern [97]. Additionally, expensive equipment is required [98].

#### Ligand-based incubation

The use of ligands on vesicles provides another method to facilitate more efficient membrane fusion during incubation. This approach involves the construction of biologically inspired structures to trigger vesicle docking and membrane fusion. One example is the use of DNA as a ligand. In a DNA-mediated membrane fusion system, single-stranded DNA is altered by hydrophobic groups to enable its spontaneous insertion into the lipid bilayer of liposomes or cells, and a second single-stranded DNA with a complementary sequence is inserted into a different liposome [99]. Although DNA-mediated membrane fusion is promising, it suffers from low fusion efficiency. Mora et al. (2020) demonstrated that vesicle fusion is facilitated by peptides (Fig. 3f). The vesicles were functionalized with lipopeptides, and the results indicated that the peptide-based membrane fusion system can be used as a rapid and effective targeted drug delivery system in future studies [100].

## Genetically engineered vesicle vector system

Genetic engineering of the parent cells is another method to modify EVs with targeted bioactive probing molecules. In the following sections, we will focus on the genetic engineering of bacterial outer membrane vesicles (OMVs) via various surface display techniques.

### Genetic engineering of EV parent cells

**Fig. 4:**
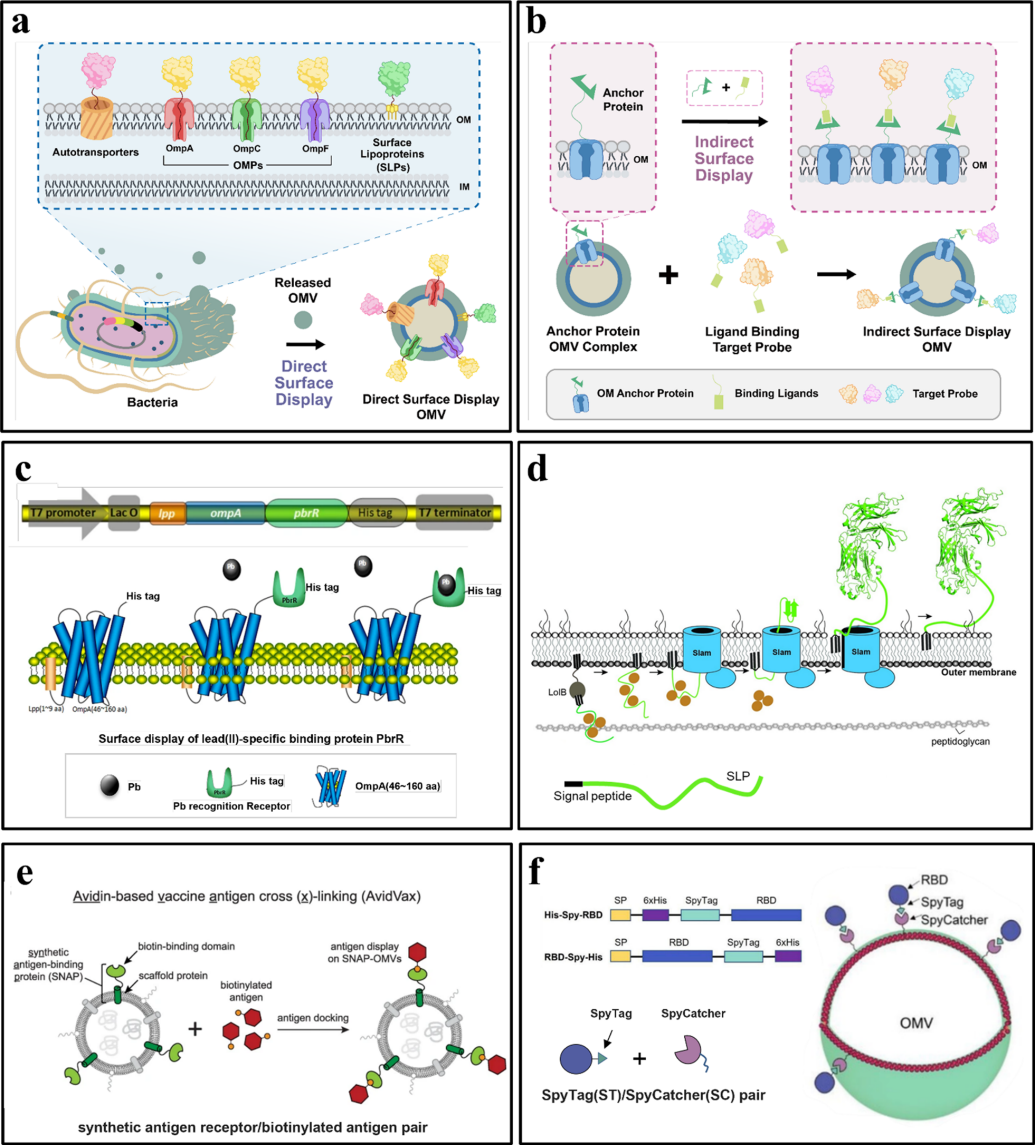
Surface Display Methods **a** Schematic illustration of the secretion of outer membrane vesicles and direct display models including autotransporters, outer membrane proteins (OMPs), and surface lipoproteins (SLPs). **b** Indirect display module. The specific bioconjugation system was utilized to produce an indirect display module between biological probing molecules and OM anchor proteins. **c** Outer Membrane Proteins. The diagram presents the cloning/ expression regions of a lead (Pb) adsorbing molecule ligated into the gene and displayed on the outer membrane of *E. coli*. Reprinted from Hui et al. 2018. **d** Slam-dependent surface lipoprotein (SLP) translocation. Reprinted from Huynh et al. 2022. **e** ClyA Fusion. The diagram depicts engineered OMVs, called AvidVax, designed for vaccine application. It displays a synthetic antigen receptor (SNAP-OMVs) that are remodeled with biotinylated antigens-of-interest using ClyA fusion technology. The surface of SNAP-OMVs can be remodeled with virtually any biomolecule amenable to biotinylation. Reprinted from Weyant et al., 2023. **f** SpyCatcher/SpyTag. Design of receptor-binding domain (RBD) recombinant antigens fused to N- and C-terminal of SpyTag and its subsequent display on the surface of OMVs. Reprinted from Jiang et al. 2022

Surface display through genetic modification allows functional proteins to be expressed on the bacterial surface by fusion with different anchor proteins [101] (Fig. 4).

Typically, displaying of recombinant proteins on EVs’ surface membrane can be achieved through a variety of established systems such as the autotransporter platform and Spy Tag/Spy Catcher system. Heterologous proteins and peptides can be directly (or indirectly) displayed on microbial cell surfaces using surface display methods that fuse proteins with anchoring motifs. Over the past three decades, several cell surface display methods have been invented, particularly for bacteria and yeast [102]. We further classified these methods into two primary categories that include direct and indirect.

Direct surface display occurs when a heterologous protein is directly displayed on the OMV surface membrane with genetically modified mediator molecules as anchors. Surface display involves the utilization of the intrinsic proteins of a bacterium to express heterologous proteins on its surface. Gram-negative bacteria use a variety of specialized protein secretion mechanisms to transport proteins across one or several phospholipid membranes [103]. The use of these secretion systems is a promising platform for the surface display of recombinant proteins on bacterial OMVs. Secretion systems such as autotransporters, outer membrane proteins (OMPs), and lipoproteins are frequently used as carriers for gram-negative bacterial surface displays, and this will be highlighted in the following sections (Fig. 4a). Conversely, indirect surface display involves the use of intermediary proteins and affinity tags such as the outer membrane anchor protein and specific binding ligand (Fig. 4b), to facilitate its display onto the OMV surface.

### Direct surface display

#### Autotransporter platform

The autotransporter (AT) system is a protein secretion pathway in Gram-negative bacteria that is used to transport large proteins across the external membrane and onto the bacterial cell surface [104]. It is a widely used cell surface display platform because AT is known to transport material from the inner membrane across the outer membrane. A typical AT protein consists of three domains, including an N-terminal signal peptide, a passenger domain, and a C-terminal translocator domain. The passenger domain that consists of a series of repetitive sequences is responsible for its cell-surface display function. These sequences then form a β-helical structure that is thought to provide stability to the protein. From a targeted delivery perspective, an autotransporter platform can be used to display ligands on the surface of OMVs to allow target specificity when delivering cargo for the treatment of certain diseases. The protein of interest was fused to the C-terminal end to use the AT platform for cell-surface display. The resulting fusion protein is then transported across the bacterial membrane by the autotransporter domain and displayed on the surface of the bacterial cells. Compared to existing cell-surface display systems, the autotransporter platform exhibits several benefits, including the capacity to display relatively larger proteins and intricate protein structures (Fig. 4a).

The applications of autotransporter platforms include the display of antigens for vaccine development, enzymes for biocatalysis, and antibodies for therapeutic purposes. Examples of autotransporter proteins used in various applications are *E. coli-*derived Ag43 [101], AIDA-I [105], and Hbp [106].

#### Outer membrane proteins

Outer membrane proteins (OMP) of Gram-negative bacteria can be used as carrier proteins for the surface display of heterologous proteins and consist of several classes. OMPs share a structural pattern with AT proteins known as beta-barrels that are composed of a variable number of transmembrane anti-parallel beta-strands joined by lengthy exterior loops and short periplasmic twists [107]. These overhanging loops are permissive locations that can accommodate foreign peptides or protein fragments, thus making them ideal carriers for surface display of heterologous proteins. Various peptides and proteins, including small molecular weight peptides, antibodies, domains, enzymes, and receptors, have been identified using the outer membrane proteins OmpA, OmpC, OmpF, OmpS, FadL, OprF, and PhoE. Hui et al*.* (2018) demonstrated the use of genetically engineered microorganisms as bioremediation adsorbents by displaying a Pb-adsorbing molecule (PbrR) using OmpA on the surface of *E. coli* [108] (Fig. 4c).

Lipoproteins (LPs) are another class of OMPs that can be used as anchors for the surface display of heterologous proteins. LPs are soluble hydrophilic proteins that remain associated with the lipid bilayer through covalently attached lipid anchors [109]. It is well established that many lipoproteins are present in the periplasm and are bound to the inner folding of the outer membrane or the outer folding of the inner membrane. These surface lipoproteins (SLPs) perform crucial functions in regard to food uptake, immunological evasion, cellular adhesion, and cell signaling and are turally and functionally varied [109]. Additionally, surface lipoproteins can be used as carrier proteins for cell-surface display of heterologous proteins [110] (Fig. 4d).

### Indirect surface display

#### ClyA fusion

Cytosolic A (ClyA) is a 34-kDa cytosolic protein produced of the most abundant gene. Biological probing molecules (e.g., proteins, peptides, carbohydrates, haptens, lipids, and aptamers) can be displayed on the surface of OMVs by acting as leading sequences that promote the localization of probing molecules onto the outer membrane. By fusion with ClyA, the gene encoding the heterologous protein produced a recombinant outer membrane protein. The extracellular expression of a biological probe molecule or protein is possible, as ClyA is attached to the outer membrane (Fig. 4e).

Cheng et al*.* displayed heterologous proteins on OMVs by fusing proteins with the ClyA protein using recombinant DNA technology [111]. To optimize the conditions for expression of the fusion proteins, the genes encoding ClyA and the indicator enzyme luciferase (ClyA-Luc, CL) were recombined in the plasmid pET28a, and the fusion protein was expressed in E. coli Rosetta (DE3). Sepahdar et al. successfully displayed an anti-EGFR affibody on the OMV surface using ClyA as an anchor protein to target triple-negative breast cancer cells with no cytotoxic, hemolytic, or immune-activating effects [112].

#### Spy catcher-Spy tag system

A Spy Catcher-Spy Tag system was designed to ligate the proteins. It is based on a modified domain of the Spy Catcher surface protein from *Streptococcus pyogenes* that can identify a cognate 13-amino-acid peptide (Spy Tag). The side chains of lysine in Spy Catcher and aspartate in Spy Tag combine to produce a covalent isopeptide connection as soon as they are recognized. This method has been applied to label proteins, produce modular vaccines, and covalently stabilize multi-protein complexes (e.g., for microscopy) [113].

The use of Spy Catcher/Spy Tag protein ligation technology to enzymatically connect antigens to hemoglobin protease that is present in high density in OMVs was described in a study by van den Berg van Saparoea et al. (2018). The membrane environment was not a hindrance to protein ligation, and this allowed for a high surface density of coupled antigens, a characteristic that has been linked to vaccine effectiveness. Effective coupling of Spy-or Snoop Catcher-nanobody chimeras to vesicles will enable targeted medication administration to tumor tissues. It would be fascinating to incorporate surface-exposed nanobodies that detect receptors unique to certain dendritic cell types while developing an OMV vaccine. This will cause certain T-cell reactions to the delivered antigens [106]. This type of modular plug-and-display OMV coupling process is flexible and reliable and enables the manufacture of drug delivery vectors with targeted properties. The Spy tag–Spy catcher technology was recently employed by Jiang et al. (2022) to create a unique, well-characterized SARS-CoV-2 vaccine candidate. The spike receptor-binding domain (RBD) obtained from mammalian cell cultures was attached to *Salmonella typhimurium* EVs. The golden Syrian hamster was used as a COVID-19 immunization model and immunized intranasally with RBD-conjugated outer membrane vesicles [114] (Fig. 4f). Their results demonstrated that OMVs are a potential alternative for future SARS-CoV-2 vaccine development against variants such as the delta/omicron variants as boosters or for certain populations due to a number of advantages of this extracellular vesicle technology.

## Challenges and limitations

Despite the potential of VVS as targeted drug delivery carriers, several concerns remain, including scale-up production, drug loading, bioavailability, storage, stability, and immunogenicity. Overcoming these hurdles requires multidisciplinary efforts to optimize EV isolation and purification techniques, develop efficient cargo loading strategies, enhance VVS stability, mitigate immunogenicity concerns, and improve targeting strategies. Even when these challenges are successfully addressed, the translation of promising in vitro and in vivo outcomes into clinical trials presents an inescapable obstacle. At present, it is of paramount importance to focus on overcoming these limitations first to unlock the complete potential of VVS-based nanocarriers, which could potentially transform targeted drug delivery and drive progress in the field of precision medicine.

In the following sections, each of the aforementioned challenges will be reviewed and the recent research efforts in overcoming them will be discussed. Furthermore, we have summarized VVS’s strengths, weaknesses, applications, and alternatives in Table 4. This reveals the advantages and disadvantages of VVS over existing technologies for targeted drug delivery.

**Table 4:** Strengths, weaknesses, applications, ang alternatives of vesicle-vector system (VVS) for targeted drug delivery

Strengths	Weaknesses
●Protection from degradation: Lipid-based vesicles *(e.g*. liposomes and extracellular vesicles) are superior vectors for drug delivery because they protect the cargo from degradation and increase its bioavailability ●Biocompatibility: The use of safe and biocompatible extracellular vesicles (EVs) for drug delivery promote cellular uptake of target cells ●Target specificity: Conjugation of bioactive probing molecules on the surface of lipid-based vesicles help target specific cells and promote targeted drug delivery ●Multi-targeted delivery: Multi-targeted delivery can be achieved by conjugating a combination of bioactive probes with different targets or by using molecules with multiple targeting ability (*e.g.* bispecific antibodies) ●Multiple approaches in designing VVS: → Conjugation of target-specific bioactive probing materials like proteins, peptides, antibodies, and aptamers with vesicles enhances targeted delivery →Hybridization of two vesicle types *(e.g.* liposome and exosome) is a promising approach in creating modified vesicles with enhanced targeted drug delivery characteristics. It takes advantage of each vesicle’s characteristics that are helpful for targeted delivery →Genetic engineering of EV’s parent cells a practical approach in displaying bioactive probing materials on EV surface to be used for targeted delivery. This can be achieved through a variety of surface display methods largely divided into two types: direct and indirect surface display	●Scale up production of EVs can be a challenge due to complexity of the isolation process and requirement of sophisticated equipment. Currently, the most widely used method for EV extraction is ultracentrifugation, but there are problems with protein contamination and low product yield ●Drug loading: In the encapsulation of drugs, current drug loading methods on EVs are still low efficiency. Additionally, the stability and long-term preservation of drug cargoes remain to be an issue ●Bioavailability: Liposomes, in general, have a low bioavailability because they are easily cleared by the immune system. Even when using PEG to evade immune response, repeated administration of PEGylated liposomes has been reported to lead to the accelerated blood clearance (ABC) phenomenon ●Storage and stability: Cell culture derived EVs cannot be kept in storage for an extended period of time. Negative 80 °C frozen storage is now the most effective all-around storage method ●Immunogenicity: Protein markers or other properties that are inherently present in EVs may be toxic and induce immune response from hosts. For instance, Lipopolysaccharide (LPS), one of the main components of OMV membranes, but it is highly immunogenic to human cells and triggers host immune response

Applications	Alternatives of VVS
●Multi-targeted delivery: multi-targeted ligands, such as bispecific antibodies can bind to or inhibit two or more targets at once, increasing the compound's therapeutic potential ●Vaccine/gene delivery VVS can be used in vaccine delivery applications. The type of vesicle and surface displayed probing material can be altered to fit the requirements for a successful vaccine/gene delivery ●Tissue regeneration: EVs are involved in the maintenance of tissue homeostasis, and they contribute to tissue repair and regeneration. For example, exosomes were reported to promote cartilage regeneration. Using an exosome-based VVS can further enhance this ability which can be applied to the regeneration of other types of tissues as well ●Immunological regulation: EVs play discrete roles in the immune regulatory functions, such as antigen presentation, and activation or suppression of immune cells. Likewise, EV-based VVS can be applied in immune regulation applications, such as for the treatment of autoimmune diseases	●Inorganic nanoparticles: Silver NP, gold NP, zinc oxide NP, and magnetic nanoparticles are used in various biomedical applications (including targeted delivery) due to their good biocompatibility, small size, low toxicity, easy surface modification, and controlled drug release ●Robotic nanomaterials: Micro/nanorobots can be designed to perform any task, including the effective delivery of drugs to body tissues. Besides targeted delivery in cancer treatment, they are also predicted to carry out other small-scale tasks, such as microsurgery of cells, assisted fertilization, and tissue engineering. Despite very promising advantages, much more research is necessary regarding micro/nanorobots on clinical applications ●Carbon nanotubes imaging probe: Carbon nanotubes have versatile applications. Its strong absorption of near-infrared regions enable their application in photothermal therapy. Carbon nanotubes can also transform the laser energy to acoustic signals and exhibit great resonant Raman scattering and photoluminescence in near infrared region, which are all beneficial to their utilization in cancer imaging

### Scale up production

EVs can be extracted either directly from bodily fluids and tissues or indirectly from cells grown in vitro. The primary obstacles to establishing EVs in clinical settings are their production, isolation, modification, and purification at scales that are appropriate for clinical use [116]. Principally, the release of EVs by cells is a spontaneous process and techniques in optimizing growth conditions to maximize EV productivity is a relatively well-studied area. Some of these techniques can be largely categorized under physical or chemical stimulation methods.

Physical stimulation mainly involves manipulation of culture conditions such as serum starvation, pH, temperature, exposure to oxygen, among others [116–119]. Sun et al*.* observed an increase in EV levels attributed to serum deprivation, along with modified size distribution and the selective enrichment of particular proteins [118]. Lower pH values also significantly enhanced EV production. These conditions led to increased uptake efficiency while maintaining cell-type specificity, presenting a notable advantage in the context of targeted delivery [119].

Chemical stimulation methods involve the simple addition of specific chemicals to the growth medium. For instance, cytochalasin-B treatment resulted in the production of at least five times more extracellular vesicle (EV) particles compared to conventional isolation methods [120]. Moreover, EVs treated with cytochalasin exhibited an enhanced capacity for drug loading [121]. Addition of calcium ions is another chemical modification used to increase EV production [122]. The addition of calcium ions in the bioreactor lead to increased intracellular ion concentration which altered the expression of proteins related to EV biogenesis. This ultimately led to an increase in EV production under calcium ion treatment conditions [123].

The technical challenge in the upscale production of EV is its isolation and purification. Currently, the most widely used methods for EV isolation and purification involves an ultracentrifugation step, which is a time consuming, operator and equipment-sensitive, and very expensive process [94]. In addition, isolation techniques that rely mainly on ultracentrifugation have no standardized protocol [124]. As a result, yield recovery and purity may largely vary depending on several key factors that determine the final efficiency of EV isolation. These factors include acceleration, type of rotor, and the inherent properties of the centrifuge like the radius of rotation and the sedimentation path length (k factor) [125].

Another limitation in scale-up production is the reproducibility of EV’s properties. The amount of EVs produced, size, as wells as its proteomic cargo largely vary according to the growth conditions of the parent cells [126, 127]. Additionally, there are problems associated with protein contamination and low yield. Therefore, methods such as density cushions ultracentrifugation have been proposed to reduce contamination by lipoproteins and plasma proteins when separating EVs [128] but effective methods such as immune isolation and microfiltration are currently preferred [117].

### Drug or cargo loading

Various loading methods have been developed for targeted drug delivery through the VVS; however, their efficiency remains low. Chen et al*.* devised Sonication and Extrusion-assisted Active Loading (SEAL) as a means to increase the drug loading and improv the transport efficiency of anticancer drugs [129]. A hybrid form of VVS using macrophages treated with DOX and iron oxide nanoparticles allowed DOX to be regulated by magnetic force when delivered to cancer cells. Additionally, attempts have been made to overcome the low solubility and absorption of curcumin by applying it to VVS. EV loading to improve the side effects and intracellular absorption of paclitaxel is an example of VVS [130]. Exosomes have been proposed as replacements for cell-based therapies, as the capabilities of the exosome cargo are becoming increasingly known. Exosomes act as carriers of RNA, lipids, and protein cargoes to alter the state of recipient cells [131]. Utilizing natural selective enrichment of the desired RNA into EVs is the primary objective of endogenous RNA loading [132]. Either a passive loading method or an active loading process can achieve this. The use of a design to overexpress the desired RNA that is subsequently loaded into EVs by the mechanism of the cell is known as passive endogenous loading [13]. This method eliminates the requirement for additional vectors that modify RNA loading through molecular interactions by using an overexpression vector to stoichiometrically increase RNA loading [133]. Preloading techniques can take some time to set up, but they offer simple and continuous production of drug-containing EVs. The integrity of the EV membrane is also preserved, as post-loading techniques that typically damage the membrane are avoided. However, as the amount of medicine loaded into EVs depends upon several variables, including transfection effectiveness and cell survival, their management can be challenging [134]. The lack of complete knowledge of the' full metabolic profile of EVs is one of the primary issues regarding their therapeutic applications. Additionally, there are no efficient drug-loading mechanisms for clinical-grade production, and there are no standardized isolation and purification techniques. Alternatives that are repeatable, inexpensive, simple, and can be modified for mass production are still required [135].

### Bioavailability

Bioavailability refers to the fraction of a drug that enters systemic circulation and is known to exert its pharmacological effect [136]. Lipid-based nanocarriers such as VVS can enhance the bioavailability of poorly soluble drugs by improving their solubility, permeability, and stability. VVS also protects drugs from enzymatic degradation, provides sustained release, and targets specific tissues and cells. However, the bioavailability of drugs delivered via VVS is limited by several factors. For example, the size, surface charge, and composition of a nanocarrier can influence its interaction with biological barriers such as the mucus layer, epithelial cells, and endothelial cells. VVS that are too large or positively charged may be trapped in the mucus layer or taken up by macrophages, thus leading to a decrease in bioavailability [137]. In contrast, VVS that are too small or negatively charged may be rapidly eliminated by the kidneys or reticuloendothelial system (RES), ultimately resulting in a short circulation time and low bioavailability [138].

Moreover, the choice of lipid and surfactant components can affect the stability and integrity of the VVS during storage, transportation, and administration [139]. Lipid and surfactant molecules can undergo oxidation, hydrolysis, or aggregation, ultimately leading to changes in the physical and chemical properties of the nanocarrier and the contained drug [140]. These changes can affect the release profile, biodistribution, and efficacy of the drug, thus limiting its bioavailability [141].

Although VVS offers promising benefits for targeted drug delivery, its bioavailability is still limited by several factors related to physical, chemical, and biological properties. Therefore, careful selection and optimization of the VVS components and parameters are crucial for achieving high drug bioavailability and therapeutic efficacy.

### Storage and stability

In recent years, several studies have investigated the storage stability of EVs and liposomes, often focusing on specific parameters such as storage temperature/time, freezing protocol, vesicle sources, or specific features [142]. The physical instability of VVS can be attributed to several factors, including membrane bilayers, aggregation, and poor retention of encapsulated contents [143]. EVs are a promising cell-free treatment; however, they cannot be stored for extended periods. Therefore, research focused on EV preservation technologies is required to safeguard their biological activity and make them practical for clinical applications and transit. Negative 80 °C frozen storage is now the most effective all-around storage method [144]. However, the choice of temperature for long-term storage stability of EVs is influenced by a variety of sources and experimental methods [17]. In recent years, a small number of studies have investigated the impact of EV sources [145] and the choice of isolation methods [146].

It is worth noting that research in the field of VVS-based drug delivery is rapidly evolving, and ongoing efforts aim to address these limitations to harness the full potential of VVSs as effective and stable drug delivery nanocarriers.

### Immunogenicity

For successful delivery, VVS must pass through multiple barriers such as the physical, immune, and (in certain cases) the blood–brain barrier before reaching the target site. Low immunogenicity allows VVS to pass through these barriers without triggering an alarm in the host immune system, thus enabling successful delivery.

Immunogenicity also significantly contributes to in vivo blood circulation time of VVS and subsequently, its bioavailability. Nanoparticles with high blood circulation time will have optimized chances of delivering its cargo into the intended target site. However, recent research in intravenous NPs has revealed that only 1% is able to reach their target sites due to immune system clearance and short blood circulation time in vivo [147, 148]. The most widely practiced technique of incorporating long-circulating and low immunogenic qualities is the addition of PEG. PEG evades the mononuclear phagocyte system by encapsulating the vesicles with a hydration layer, thus giving the VVS a “stealth” property [149, 150]. However, upon continued administration, hosts can develop accelerated blood clearance (ABC phenomenon) wherein the nanoparticle-carrying drugs are rapidly removed from the blood and delivered to the liver and spleen causing toxicity in the liver and spleen [151]. Recently, a new approach in the development of VVS has been developed by Fang *et. al.* They demonstrated the use of RBC-derived membrane materials to disguise drug nanocarriers as ‘self’, resulting in significantly prolonged blood circulation time that is even greater compared to its PEGylated counterparts [152].

Immunogenicity of VVS as drug delivery nanocarriers is a complex and multifactorial issue, and further research is needed to fully understand and address these limitations.

### Clinical applications

VVS has exhibited effective targeted drug delivery abilities both in vitro and in vivo. However, the transition from these promising results to practical clinical applications poses a significant challenge. Up to this point, VVS in the form of PEGylated liposomes, notably demonstrated by Doxil® and Onivyde® in the context of cancer treatment, have been the only treatments to achieve substantial success in clinical trials, ultimately receiving FDA approval [153]. Nevertheless, their clinical utility remains constrained by a lack of precise targeting. Moreover, repeated administrations of PEGylated VVS resulted in the emergence of the accelerated blood clearance (ABC) phenomenon [154].

In contrast to liposomes, EVs inherent qualities—role in cell-to-cell communication, biocompatibility, low immunogenicity, stability, and the ability to cross biological barriers—make them excellent candidates for clinical applications as diagnostic biomarkers and as targeted drug delivery vectors for the treatment of cancer and other immunological diseases [155]. However, recent clinical trials using VVS for drug delivery have mainly used unmodified EVs, which has raised concerns related to lack of target specificity, low EV half-life, heterogeneity, dependence on parent cell condition, and the cost associated with large-scale cell culture [156, 157]. Nonetheless, a few emerging pharmaceutical companies are pushing efforts to bring the use EV-based VVS into clinical applications [158]. In particular, Codiak Biosciences made a VVS called ExoIL-12™ for the targeted delivery of drugs in cancer treatment [159]. They have engineered exosomes to display interleukin-12 (IL-12) by using Prostaglandin F2 Receptor Negative Regulator (PTGFRN), a type I membrane protein abundantly expressed on the exosome surface, serving as an anchor to display IL-12 on the surface of exosome membrane [159]. Phase I of clinical trials has demonstrated a promising safety and tolerability profile in repeated dosing, along with observed antitumor activity in both locally injected and distant lesions [160]. Consequently, the recommendation for phase 2 trials is based on these positive outcomes, with optimistic expectations for continued success.

In conclusion, the promising potential of VVS for targeted delivery in clinical trials emphasizes the need for continued investigation and research, paving the way for advancements in this field.

### VVS Applications, insights, and future perspectives

VVS can be modified and applied in a variety of fields in biotechnology and medicine in addition to their use in targeted drug delivery.

VVS is a favorable platform for vaccine and gene delivery. State-of-the-art vaccine delivery systems involve the encapsulation mRNA-based vaccines with lipid-based nanovesicles [139]. The same concept has also been used for gene delivery. Numerous papers have demonstrated the successful application of EVs as vehicles for nucleic acids due to their easy cargo loading (via electroporation) and transfer of nucleic acid cargo into mammalian host cells [161, 162]. However, stronger pulses may cause harm to both the EVs and their cargo, and electroporation is linked to nucleic acid species aggregation and EV membrane integrity loss. However, it has been demonstrated that altering the electroporation settings might lessen these undesirable consequences [163]. Further modification of liposomes and EVs into VVS will improve the target specificity and increase the overall efficacy. In addition to encapsulation, the surface decoration of antigens provides another promising approach for vaccine delivery. VVS vaccines can be produced using surface-decorating EVs with specific antigens that trigger an immune response. Surface-attached antigens exhibit an advantage in inducing a T-cell response due to their availability for B-cell recognition as opposed to encapsulated antigens that require vesicle disruption to be accessible [164]. A promising approach to VVS-based vaccines would be to impede viral infection via surface decoration of VVS with virus-host interaction-inhibiting molecule, such as Cyclophilin A [165].

Therefore, the therapeutic potential of VVS requires further exploration. EVs participate in maintaining tissue homeostasis, repair, and regeneration [166, 167]. Specifically, exosomes promote cartilage regeneration [168]. This ability can be further exploited using exosome-derived VVS and can be applied in the fields of artificial cell culture and tissue regeneration. Related to tissue regeneration, exosomes (derived from bovine milk) were also reported to help reduce scars or keloids, caused by abrasion, skin tissue damage, skin incision by surgery, and acne extrusion in vitro [19]. VVS based on exosomes can be further explored for scar-free healing treatments and applications. Additionally, EVs play diverse roles in the regulation of the immune system, including antigen transport and suppression and activation of immune cells [169]. Similarly, EV-based VVS can be used in immune regulation applications such as the treatment of autoimmune diseases.

According to recent research, EVs are crucial in the transfer of autoantigenic peptides from β cells that produce insulin during the onset of type 1 diabetes (T1DM). T1DM patients' plasma had higher levels of expression of miRNAs in EVs, such as miR-16-5p, miR-574-5p, and miR-302d-3p, than did the plasma of healthy controls [170]. Research has demonstrated that administering pure srIκB-loaded exosomes (Exo-srIκBs) intraperitoneally reduces mortality and systemic inflammation in a mouse model of sepsis [171]. Additionally, in vitro and in vivo studies have shown that the inclusion of phytochemicals into EVs as part of nanomedicine for anticancer therapy is effective. The chemical stability, bioavailability, and site activity of the integrated phytochemicals are improved by EV delivery of anticancer phytochemicals [172]. Discovered tumor growth suppression in an orthotopic mouse model of breast cancer by encapsulating miRNA-379 into mesenchymal stem cell (MSC)-derived extracellular vesicles (EVs) through the transfection of MSCs with miRNA-379 [173]. A study used exosomes produced from IFN-γ-modified RM-1 prostate cancer cells in C57BL with lung metastases to provide a vaccination schedule consisting of four injections (days 0, 4, 8, and 12). In male mice, it decreased the quantity of Tregs and the tumor metastatic rate [174]. These technologies provide a potential and feasible personalized exosome-based cancer vaccine approach produced from tumors and dendritic cells [175]. EV applications can also extend to addressing bacterial infections in the context of hospital settings. For example, exosomes derived from honey were discovered to contain antimicrobial peptides effective against oral *Streptococci*. It showed antibacterial and antibiofilm capabilities which were notably more pronounced on *S. mutans* in comparison to *S. sanguinis* [176]. Likewise, bovine colostrum-derived exosomes (BC-Exo) also showed biofilm eradication and anti-hemolysis effects against *Staphylococcus aureus*. A decrease in ATP production and modification of the cell surface due to BC-Exo treatment were observed. The antibacterial activity of BC-Exo against *S.aureus* was confirmed, and this can be a base for the development of antibiotics [177].

Despite its obvious merits, VVS remains a competitive drug delivery platform. Inorganic nanoparticles (INP) such as AgNPs, AuNPs, ZnONPs, and magnetic NP have been explored and applied in the fields of biotechnology and medicine as drug delivery vehicles since the 1960s due to their low toxicity, small size, biocompatibility, ease of surface modification, and potential in the facilitation of controlled drug release [178]. Another consideration regards its use (CNT). CNTs exhibit similar advantages to INPs; however, due to their distinctive needle-like structure, they can easily penetrate cell membranes [179]. Robotic nanomaterials are an emerging technology that can be used as drug delivery vectors. Nanorobots can be designed to perform any task, including effective delivery of drugs to body tissues. In addition to targeted delivery, they are also predicted to perform other micro/nanoscale tasks such as cell tissue engineering, assisted fertilization, and microsurgery [180].

However, future research directions for VVS are limitless. It must be further explored for applications not only in targeted delivery but also in all other areas where it may serve a significant purpose.

## Conclusion

Targeted delivery using well-constructed nanocarriers such as the vesicle-vector systems (VVS) can be used to treat numerous illnesses with enhanced efficacy and reduced adverse effects. In this review, we introduced VVS as a flexible carrier for targeted drug delivery with great potential to also be applied in the areas of vaccine delivery, gene therapy, and tissue regeneration. We specified three different approaches for the development of VVS—vesicle-probe, vesicle-vesicle, and genetically engineered vesicles—and showed evidence of the improved target-specificity for the delivery of diverse therapeutic materials. However, the recently developed VVS is still far from achieving the ultimate goal of the “magic bullet”. Investigations examining the pharmacokinetic profile of loaded drugs and their biodistribution on vesicles upon loading and release in vivo and in actual clinical settings remain to be a major concern. Hence, studies involving in vivo testing and clinical trials should be given consideration. By highlighting the vesicle vector system (VVS) and its potential applications in targeted drug delivery, we hope to encourage and inspire the readers to explore new methods to overcome its limitations and create new approaches in developing cost-effective, safe, and effective targeted drug delivery systems hereinafter.

## Data Availability

Data availability is not applicable to this article as no new data were created in this study.

## Abbreviations

VVS: Vesicle-vector system
DDS: Drug delivery system
NP: Nanoparticle
OMV: Outer membrane vesicle
EV: Extracellular vesicle
PEG: Polyethylene glycol
CS: Chitosan
RES: Reticuloendothelial system
HUVEC: Human umbilical vein endothelial cells
MSC: Murine mesenchymal stem cell line C3H
MDCK (free GFP expressing): Mardin−Darby canine kidney cells
LPS: Lipopolysaccharides
BsAbs: Bispecific Antibodies
INP: Ice nucleation proteins
DOPE: 1,2-Dioleoyl-sn-glycero-3-phosphoethanolamine
DOTAP: 1,2-Dioleoyl-3-trimethylammonium-propane (chloride salt)
HSPC: L-a-phosphatidylcholine
DOPC: 1,2-Dioleoyl-sn-Glycero-3-Phosphocholine
POPC: 1-Palmitoyl-2-oleoyl-sn-glycero-3-phosphocholine
DMPC: 1,2-Dimyristoyl-sn-glycero-3-phosphocholine
DSPE: 1,2-Distearoyl-sn-glycero-3-phosphorylethanolamine
DSPA: 1,2-Distearoyl-sn-Glycero-3-Phosphatidic acid Na salt
DSPG: 1,2-Distearoyl-sn-glycero-3-phosphorylglycerol sodium salt
DSPC: 1,2-Distearoyl-sn-glycero-3-phosphocholine
DPPE: 1,2-Dipalmitoyl-sn-glycero-3-phosphorylethanolamine
DPPA: 1,2-Dipalmitoyl-sn-glycero-3-phosphatidic acid sodium salt
DPPG: 1,2-Dipalmitoyl-sn-glycero-3-phosphorylglycerol sodium salt
DPPC: 1,2-Dipalmitoyl-sn-glycero-3-phosphocholine (DPPC)
OMP: Outer Membrane Proteins
SLP: Surface Lipoproteins
